# Legal Information Retrieval and Entailment Based on BM25, Transformer and Semantic Thesaurus Methods

**DOI:** 10.1007/s12626-022-00103-1

**Published:** 2022-02-07

**Authors:** Mi-Young Kim, Juliano Rabelo, Kingsley Okeke, Randy Goebel

**Affiliations:** 1 Department of Science, Augustana Faculty, University of Alberta, Camrose, AB Canada; 2 Alberta Machine Intelligence Institute, University of Alberta, Edmonton, AB Canada

**Keywords:** Legal Information Extraction, Legal Information Entailment, BM25, Transformers, 68T50, 68T07, 68T05

## Abstract

We describe the techniques applied by the University of Alberta (UA) team in the most recent Competition on Legal Information Extraction and Entailment (COLIEE 2021). We participated in retrieval and entailment tasks for both case law and statute law; we applied a transformer-based approach for the case law entailment task, an information retrieval technique based on BM25 for legal information retrieval, and a natural language inference mechanism using semantic knowledge applied to statute law texts. This competition included 25 teams from 14 countries; our case law entailment approach was ranked no. 4 in Task 2, the BM25 technique for legal information retrieval was ranked no. 3 in Task 3, and the natural language inference technique incorporating semantic information was ranked no. 4 in Task 4. The combination of the latter two techniques on Task 5 was ranked no. 2. We also performed error analysis of our system in Task 4, which provides some insight into current state-of-the-art and research priorities for future directions.

## Introduction

Tools to help legal professionals manage the increasing volume of legal documents are now essential. The volume of information produced in the legal sector by its many different actors (e.g., law firms, law courts, independent attorneys, legislators, and many other sources) is overwhelming. To help build a legal research community, the Competition on Legal Information Extraction and Entailment (COLIEE) was created, to develop a research community that focuses on four specific challenge problems in the legal domain: case law retrieval, case law entailment, statute law retrieval and statute law entailment. Here we provide details of our approaches for the legal information retrieval and legal text entailment tasks.

The competition began in 2014, and completed its eighth edition this year. Over its history, initial techniques for open-domain textual entailment focused on exploiting shallow text features. But after eight years and competition and discussion amongst many teams has evolved the choice of methods to include the usage of word embeddings, logical models and general machine learning. The current state-of-the-art, especially for problems which have access to enough labeled data, relies on deep learning-based approaches (more notably those based on transformer methods), which have shown very good results in a wide range of textual processing benchmarks, including benchmarks specific to entailment tasks.

Our method for the case law entailment task is based on adapting our methods from the past editions [1, 2], with an increased focus on transformer methods and a heuristic post-processing technique based on *a priori* probabilities. In this year, we decided to drop similarity calculations, as our previous results have shown they did not significantly contribute to improved performance. For the statute law tasks, we applied Opkapi Best Matching otherwise called “BM25,” for the retrieval task and a combination of a transformer-based methods and exploitation of semantic information for the entailment tasks. In the future, we intend to further explore techniques to capture semantic similarity and experiment with data augmentation methods.

The rest of this paper are organized as follows: in Sect. 2, we briefly review information retrieval (IR) and textual entailment. Section 3 describes our current methods and presents our results on both case law and statute law entailment tasks in COLIEE 2021. Section 4 concludes the paper and comments on future work.

## Related Work

Textual entailment, which is also called Natural Language Inference (NLI), is a logic task in which the goal is to determine whether one sentence can be inferred from another (more generally, whether one text segment can be inferred from another).

In the sentential case, the task consists of classifying an ordered pair of sentences into one of three categories: “positive entailment” occurs when one can use the first sentence to prove that a second sentence is true. Conversely, “negative entailment” occurs when the first sentence can be used to disprove the second sentence. Finally, if the two sentences have no correlation, they are considered to have a “neutral entailment.” In COLIEE, teams are challenged with the task of determining whether two case law textual fragments have a “positive entailment” relationship or not (i.e., either “negative entailment” or “neutral entailment”). The statute law entailment task (Task 4) in COLIEE is similarly designed: the participants are required to decide if a query is entailed from the texts of relevant civil law statutes.

In the following subsections, we will discuss related research on textual entailment in general, and the specific techniques we have developed for case law entailment.

### Open-Domain Textual Entailment

Textual entailment can be viewed as an independent task per se or as a component in larger applications. For example, question–answering systems may use textual entailment to identify an answer from previously stored answer databases [3]. Textual entailment may also be used to enhance document summarization (e.g., used to measure sentence connectivity or as additional features to summary generation [4]). Because of growing interest in textual entailment, there has been an increase in publicly available benchmarks to evaluate such systems (e.g., [5, 6]).

Early approaches for open-domain textual entailment relied heavily on exploiting surface syntax or lexical relationships, which have subsequently been elaborated with a broader range of tools, such as word embeddings, logical models, graphical models, rule systems and machine learning [7]. A modern research trend for open-domain textual entailment is the application of general deep learning models, such as ELMo [8], BERT [9] and ULMFit [10].

These methods build on the approach introduced by Dai and Le [11], which showed how to improve document classification performance using unsupervised pre-training of an LSTM [12], followed by supervised fine-tuning for domain specific downstream tasks. The pre-training is typically done on very large datasets, which do not need to be labeled and are intended to capture general language use knowledge like co-occurrence of words. This pre-training is usually formulated as a language modeling task. Subsequently, supervised learning can be used as a fine-tuning step, thus requiring a labeled but significantly smaller dataset, which aims to adjust the weights of the final layers of the model suitable for a specific task. These models have achieved impressive results in a wide range of publicly available benchmarks of different common natural language tasks, such as RACE (reading comprehension) [13] , COPA (common sense reasoning) [14] and RTE (textual entailment) [15], to name a few.

### Case Law Textual Entailment

The specific task of assessing textual entailment for case law documents is quite new. The first COLIEE edition to include this task was in 2018 [16]. In that competition, Chen et al. [17] proposed the application of association rules for the problem. They applied a machine learning-based model using Word2Vec embeddings [18] and Doc2Vec [19] as features. This approach faces two main problems: the lack of sufficient training data to make the models converge and generalize, and the computational cost of training, which increases exponentially on the size of the dataset. To overcome that issue, they proposed two association rule models: (1) the basic association rule model, which considers only the similarity between the source document and the target document, and (2) the co-occurrence association rule model, which uses a relevance dictionary in addition to the basic model.

Another technique [20] worth mentioning approached the task as a binary classification problem, and built feature vectors comprised of the measures of similarity between the candidate paragraph and (1) the entailed fragment of the base case, (2) the base case summary and (3) the base case paragraphs (actually a histogram of the similarities between each candidate paragraph and all paragraphs from the base case). These feature vectors are the used as input to a Random Forest [21] classifier. To overcome the problem of severe data imbalance in the dataset, the dominant class was under-sampled and the rarer class was over-sampled by SMOTE sample synthesis [22].

Since COLIEE 2019, techniques based on BERT or other transformer -based models have dominated the COLIEE case law entailment task. Rabelo et al. [1] present a method for case law entailment combining similarity-based features which rely on multi-word tokens instead of single words, and exploited the BERT framework [9], fine-tuned to the task of case law entailment on the provided training dataset. In 2020, the task 2 winner [23] applied an approach which has a model capturing the supporting relation of a text pair, based on the BERT base model, then fine-tuned for a supporting text-pair classification task. The set of supporting text-pairs includes the text-pairs from Task 1 candidate cases using designed heuristics, and the gold standard data of Task 2 (decision-paragraph). This system also uses a BERT model fine-tuned on SigmaLaw (a law dataset described in [24]) for the masked language modeling task. Together with scoring by the BERT models, lexical matching (BM25) is also considered for predicting decision-paragraph entailment. Other teams have used BERT to generate features that are then input to other classifiers. For example, Alberts et al. [25] applied an Xgboost classifier with the following features as input: NLI probability (bert-nli), similarity between entailed fragment and paragraphs based on fine-tuned BERT (bert-base-uncased), and BM25 similarity between entailed fragment and paragraphs. Those authors also submitted runs using other features as input: n-grams, BM25, NLI, and EUR-LEX (81,000 sentences from EU legal documents) fine-tuned ROBERTA and BERT (bert-base-uncased) derived similarity features.

### Statute Law Textual Entailment

Natural language inference (NLI), the task of identifying whether a hypothesis can be inferred from, contradicted by, or not related to a premise, has become one of the standard benchmark tasks for natural language understanding. NLI datasets are typically built by asking annotators to compose sentences based on premises extracted from corpora, so that the composed sentences stand in entailment/contradiction/neutral relationship to the premise [26]. In COLIEE 2021, we have two relationships that need to be verified: entailment and non-entailment. Yang et al. [27] showed that human-created knowledge can further complement the use of pre-training models, to achieve better NLI prediction. Based on the results of Yang et al. [27], we have exploited the external knowledge of the Kadokawa thesaurus [28], to tackle Tasks 4 and 5.

For information retrieval, Shan et al. [29] claimed that empirical studies showed global representative features like BM25 can capture term importance with global context information. A word with a high BM25 score reveals its uniqueness in the corpus, and this method has been widely adopted in traditional learning to rank tasks.

## COLIEE 2021—Approaches and Results

Legal question answering can be considered as a number of intermediate steps. For instance, consider a question such as “The landowner shall have the owner of the adjacent land repair or remove the obstacle if the owner of the adjacent land is damaging his or her land due to the destruction or blockage of the drainage ditch installed in the adjacent land?” In this example, a system must first identify and retrieve relevant documents, typically legal statutes. It must then compare the semantic connections between the question and the relevant legal statutes, and determine whether an entailment relation holds.

COLIEE includes both retrieval and entailment tasks in two broad areas: case law and statute law. The case law retrieval task consists in determining which cases from a pool should be “noticed” with respect to each base case in a given list. The entailment task for case law consists in determining whether an entailment relationship exists between one or more paragraphs in a referenced case and a given fragment from a base case. Note that the general idea is to identify these fragments as proxies for an overall legal argument based on noticed cases, not that any single fragment is necessary and sufficient for a complete legal case argument.

**Fig. 1:**
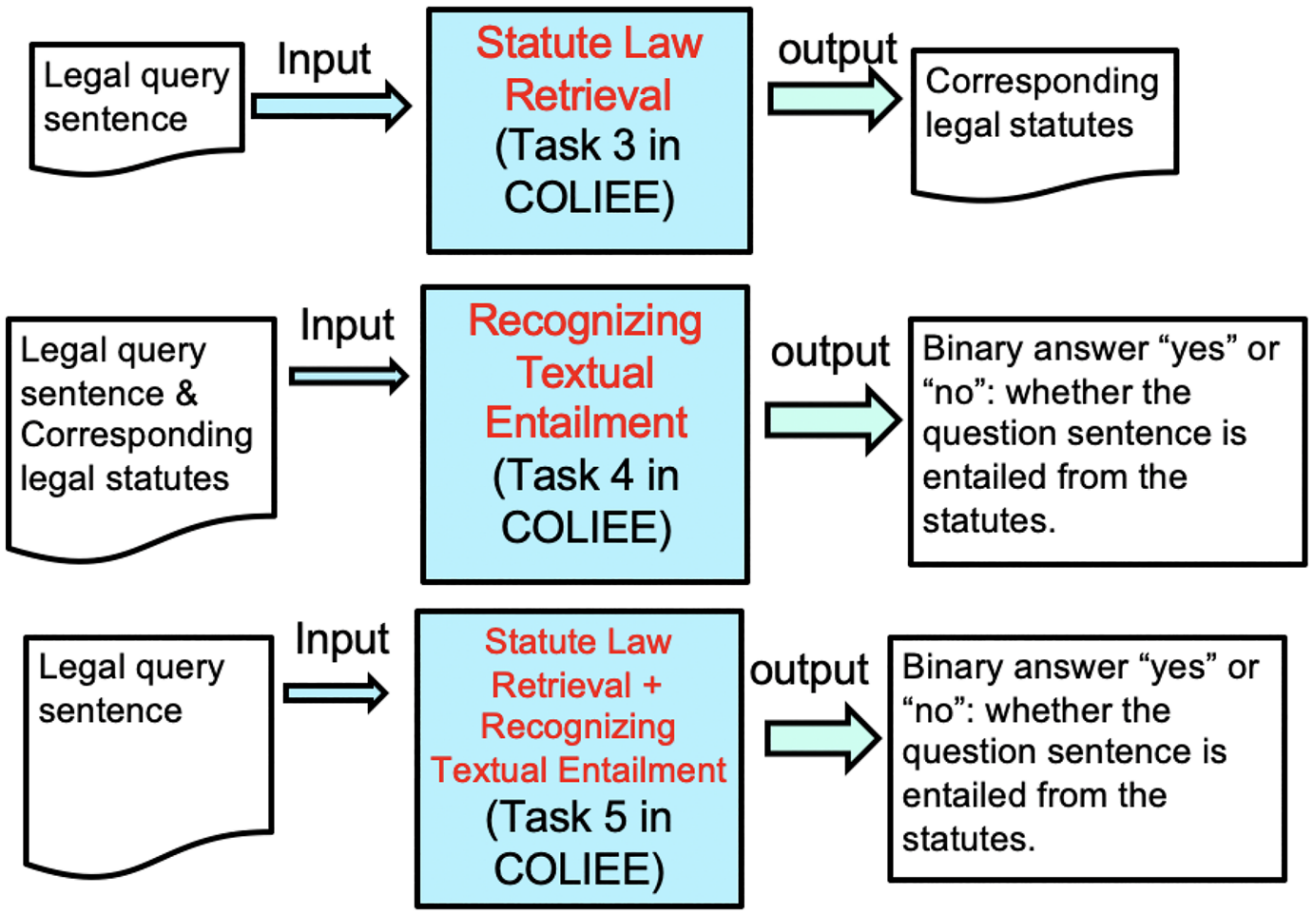
Architectures of Tasks 3, 4 and 5

For case law, the competition focuses on two aspects of legal information processing: case law retrieval (Task 1), and case law entailment (Task 2). For statute law, the competition provides three aspects of legal information processing related to answering yes/no questions from legal bar exams: legal document retrieval (Task 3), natural language inference (NLI) for Yes/No question answering of legal queries (Task 4), and a combination of document retrieval and natural language inference (Task 5). Figure 1 shows the architectures of Tasks 3, 4 and 5.

In the next subsections, we present further details on the methods we have developed and applied in COLIEE 2021.

### Case Law Entailment—Task 2

#### Task Definition

Task 2 is a legal case entailment task and it requires the identification of a paragraph from existing cases that can be claimed to entail the decision of a new case. Given a decision Q of a new case and a relevant case R, the challenge is to identify a specific paragraph in R that entails the decision Q. The organizers have confirmed that the answer paragraph cannot be identified merely by information retrieval techniques using some examples. Because the case R is a relevant case to Q, and many paragraphs in R could be relevant to Q, regardless of whether any one paragraph supports the required entailment. This task requires one to identify a paragraph which entails the decision of Q, so required is a specific entailment method that compares the meaning of each paragraph in R and the decision in Q. The data are drawn from an existing collection of predominantly Federal Court of Canada case law documents. The evaluation measures are based on information retrieval measures: precision, recall and F-measure.

#### Approach

The main component of our case law entailment method applies BERT [9] by fine-tuning on the provided training dataset. BERT is a framework designed to pre-train deep bidirectional representations by jointly conditioning on both left and right contexts in all layers. This leads to pre-trained representations which can be fine-tuned with only one additional output layer on downstream tasks, such as question answering, language inference and textual entailment, but without requiring task-specific modifications. BERT has been used to achieve very good results on other well-known benchmarks, such as GLUE [6], MultiNLI [30] and MRPC [31].

We used a BERT model pre-trained on a large (general purpose) dataset (the goal being make it acquire general language “knowledge”^1^) which can be fine-tuned on smaller, specific datasets (the goal being to make it learn how to combine the previously acquired knowledge in a specific scenario). This makes BERT a good fit for this task, since we do not have a large dataset available for training the model. Our BERT model is based on the HuggingFace uncased-BERT distribution (bert-base-uncased), then fine-tuned on the COLIEE training dataset for 3 epochs (remaining hyperparameters used as default), using input pairs of entailment fragment and candidate paragraph, then confirming whether or not there is an entailment relationship.

We encode each candidate paragraph and its corresponding entailed fragment. If the tokenization step produces more than the 512 token limit, we apply another transformer-based model [32] to generate a summary of the input text, and then process the pair again. Since the input text often includes text in French, we remove those fragments by applying a simple language detection model^2^ based on a naive Bayes filter.

The fine-tuned model is then applied to the test dataset (with the same summarization model, when needed). The model predicts scores for the entailment and non-entailment classes, which are later used in post processing the results. The objective of the post-processing step is to add some context to the classification: the classifier itself only sees pairs of input candidate paragraphs and entailed fragments, so it could easily output a high score for many of those candidates in the same case or not produce any one with a high enough score for a different case. Whether those situations are potentially feasible, the priors show that usually there are very few actual entailing paragraphs in a case (by far, most of the cases only have one entailing paragraph). So in the post-processing step, we establish limits for the maximum number of outputs allowed per case. At the same time, we know at least one paragraph is the “correct” answer. We also make use of that fact to expect that at least one paragraph should be returned, but in this case, we do use an empirically determined minimum score in an attempt to reduce number the of false positives.

Because pre-training influences how transformer-based models “understand” language, we decided to experiment with LegalBERT [33], which is a BERT model fine-tuned on legal corpora. Our assumption was that a model trained on a large legal corpus would provide better results in a legal classification task such as the case law entailment in COLIEE. The LegalBERT model was fine-tuned using the same procedure described for the generic BERT model (please see above), but the final results produced were disappointing, with a very low f1-score in a validation dataset. Despite these results, we intend to further explore this option in future editions of COLIEE as we understand pre-training transformer-based models using same domain text have the potential to provide good results. The pre-trained LegalBERT model used in our experiments is available at HuggingFace (model id: ’nlpaueb/legal-bert-base-uncased’).

As previously mentioned, in past editions, we tried to expand the training dataset through data augmentation techniques based on back translation (English to German to English) but that did not produce the expected improvements. We speculate that back-translation methods do not generate enough variability in the new examples and contribute only some slight perturbation around the existing data points. Nevertheless, we intend to further explore the data augmentation idea in future editions, but experiment with different techniques. One of the potential data augmentation techniques would rely on the (larger) dataset provided for Task 1 (case law retrieval): we intend to increase size of the training dataset by extracting simple examples of entailment relationship through hand written heuristic rules and adding those examples to the training set for Task 2. We hypothesize that simple text augmentation techniques such as synonym replacement will not provide enough variation over the existing data and do not intend to explore those options.

The official COLIEE 2021 results for this task are shown in Table 1. Our submissions were based on the fine-tuned BERT model with summarization enabled for long paragraphs and entailed fragments as detailed above. The difference between the submissions are in the post-processing parameters: UA_reg_pp.txt applies a post-processing which will keep at most one answer per case given its confidence score is at least -1. UA_def_pp.txt is similar but requires the minimum confidence score to be at least 0. UA_loose_pp.txt also established 0 as the minimum score but allows for at most 2 predictions to be made for each base case.

**Table 1:** Task 2 official results

Team	File	F1
NM	Run_task2_DebertaT5.txt	0.6912
NM	Run_task2_monoT5.txt	0.6610
NM	Run_task2_Deberta.txt	0.6339
UA	UA_reg_pp.txt	0.6274
JNLP	JNLP.task2.BM25Sup._Den..txt	0.6116
JNLP	JNLP.task2.BM25Sup._Den._F..txt	0.6091
UA	UA_def_pp.txt	0.5875
JNLP	JNLP.task2.NFSP_BM25.txt	0.5868
siat	siatCLS_result-task2.txt	0.5860
DSSIR	run_test_bm25.txt	0.5806
siat	siatFGM_result-task2.txt	0.5670
UA	UA_loose_pp.txt	0.5603
TR	task2_TR.txt	0.5438
DSSIR	run_test_bm25_dpr.txt	0.5161
DSSIR	run_test_dpr.txt	0.5161
MAN01	[MAN01] task2 run1.txt	0.5069
MAN01	[MAN01] task2 run0.txt	0.2500

### Statute Law Information Retrieval—Task 3

#### Task Definition

Task 3 requires the retrieval of an appropriate subset ($S_1$, $S_2$,..., $S_n$) of Japanese Civil Code Articles from the Civil Code texts dataset, used for answering a Japanese legal bar exam question *Q*.

An appropriate subset means the identification of a subset of statutes for which an entailment system can judge whether the statement *Q* is true $Entails(S_1, S_2, ..., S_n , Q)$ or not $Entails(S_1, S_2, ..., S_n , \lnot Q)$.

#### Approach

The key component of the probabilistic information retrieval (IR) model is to estimate the probability of relevance of the documents for a query. This is where most probabilistic models differ from one another. A number of weighting formulae have been developed and BM25 [34] has, so far, been the most effective. The major differences between BM25 and the other commonly used TFIDF models are the slight variants of inverse document frequency (IDF) formulation and the use of the query term frequency. TFIDF is computed as following:

$$\begin{aligned} tfidf(D,Q)=\sum _{t}[\sqrt{tf(t,D)}*(1+log(idf(t)))^2] \end{aligned}$$

where *D* is a document, *Q* is a query, and *t* is a term in *Q*. Here, *tf*(*t*, *D*) is *t*’s term frequency in the document *D*, and *idf*(*t*) is the inverse document frequency of *t*.

The length normalization factor in BM25 uses the average document length and a parameter has been introduced to control the relative length effect. A probabilistic language modeling technique [35, 36], is another effective ranking model that is widely used. Typically, language modeling approaches compute the probability of generating a query from a document, assuming that the query terms are chosen independently. Unlike TF-IDF models, language modeling approaches do not explicitly use document length factor and the IDF component. It seems that the length of the document is an integral part of this formula and that automatically takes care of the length normalization issue [37]. However, smoothing is crucial and it has very similar effect as the parameter that controls the length normalization components in pivoted normalization or the BM25 model. Three major smoothing techniques (Dirichlet, Jelinek-Mercer and Two-stage) are commonly used in this model [38], and we use Dirichlet smoothing in our language model-based IR [36] for COLIEE 2021. We use the following language model-based information retrieval formula:

$$\begin{aligned} {\hat{p}}(Q|M_{d})=\prod _{w\in Q}p(w|M_{d})*\prod _{w \notin Q}(1.0-{\hat{p}}(w|M_{d})) \end{aligned}$$

Here *Q* is a query, *d* is a document, and $M_{d}$ is a language model of *d*. We would like to estimate ${\hat{p}}(Q|M_{d})$, the probability of the query *Q* given the language model of document *d* as shown in the equation above. For more details on each probability such as ${\hat{p}}(w|M_{d})$, see Ponte and Croft [36].

BM25 is computed as following:

$$\begin{aligned} score(D,Q)=\sum _{i=1}^{n}IDF(q_i)\frac{f(q_i, D) * (k_1+1)}{f(q_i,D)+k_1 * (1-b+b*\frac{|D|}{avgdl})} \end{aligned}$$

where $f(q_i, D)$ is $q_i$’s term frequency in the document *D*, |*D*| is the length of the document *D* in words, and *avgdl* is the average document length in the text collection from which documents are drawn. $K_1$ and *b* are free parameters. We used 1.5 for $K_1$ and 0.75 for *b*. $IDF(q_i)$ is the *IDF* (inverse document frequency) weight of the query term $q_i$. It is usually computed as:

$$\begin{aligned} IDF(q_i)=ln\left(\frac{N-n(q_i)+0.5}{n(q_i)+0.5}+1\right) \end{aligned}$$

where *N* is the total number of documents in the collection, and $n(q_i)$ is the number of documents containing $q_i$^3^.

The legal IR task that we use to test our system has several sets of queries paired with a subset of Japan civil law articles as documents (724 articles in total). Here follows one example of the query and a corresponding relevant article:

*Question: Land owners can cut off the branches of bamboo trees in the neighboring land when they cross the border.*

*Related Article: Article 233 (1) When a branch of a bamboo tree in the adjacent land crosses the boundary line, the owner of the bamboo tree may cut the branch. (2) When a branch of a bamboo tree in the adjacent land crosses the boundary line, the owner of the bamboo tree may cut the branch.*

Before the final test set was released, we received 14 sets of queries for a “dry run” in COLIEE 2021. The 14 sets of data include 806 queries, and 1040 relevant articles (average 1.29 articles per query). The metrics for measuring our IR model performance is F2:

$$\begin{aligned} F2=\frac{5 * Precision * Recall}{4 * Precision + Recall} \end{aligned}$$

Table 2 shows the results of experiments with our three IR models on the final test set in COLIEE 2021: BM25 (BM25.UA), TF-IDF (TFIDF.UA), and language-model-based IR (LM.UA). BM25 showed the best performance amongst the three models. The test data size is 81 queries for Task 3. The performance of our system was ranked 3rd among the submitted systems in the Competition on Legal Information Extraction/Entailment (COLIEE) 2021.

**Table 2:** IR (Task3) results on test run data in COLIEE 2021

Team	F2	P	R	MAP	R_5	R_10	R_30
OvGU_run1	0.73	0.67	0.77	0.74	0.75	0.81	0.85
JNLP.CLMLT	0.72	0.60	0.80	0.79	0.78	0.89	0.95
BM25.UA	0.70	0.75	0.70	0.75	0.71	0.73	0.81
JNLP.CLBJP	0.70	0.62	0.77	0.77	0.82	0.84	0.90
R3.LLNTU	0.70	0.66	0.74	0.78	0.79	0.83	0.91
R2.LLNTU	0.70	0.67	0.73	0.78	0.78	0.84	0.91
R1.LLNTU	0.68	0.63	0.73	0.78	0.78	0.84	0.91
JNLP.CLBJ	0.68	0.55	0.77	0.77	0.81	0.84	0.91
OvGU_run2	0.67	0.48	0.80	0.75	0.75	0.81	0.90
TFIDF.UA	0.65	0.67	0.65	0.73	0.72	0.74	0.81
LM.UA	0.54	0.56	0.54	0.64	0.64	0.68	0.81
TR_HB	0.52	0.33	0.61	0.66	0.71	0.74	0.84
HUKB-3	0.52	0.29	0.69	0.61	0.68	0.74	0.87
HUKB-1	0.47	0.23	0.65	0.61	0.66	0.75	0.87
TR_AV1	0.35	0.26	0.51	0.46	0.43	0.47	0.56
TR_AV2	0.33	0.14	0.55	0.43	0.39	0.44	0.49
HUKB-2	0.32	0.32	0.32	0.41	0.46	0.54	0.61
OvGU_run3	0.30	0.15	0.70	0.55	0.57	0.61	0.70

**Fig. 2:**
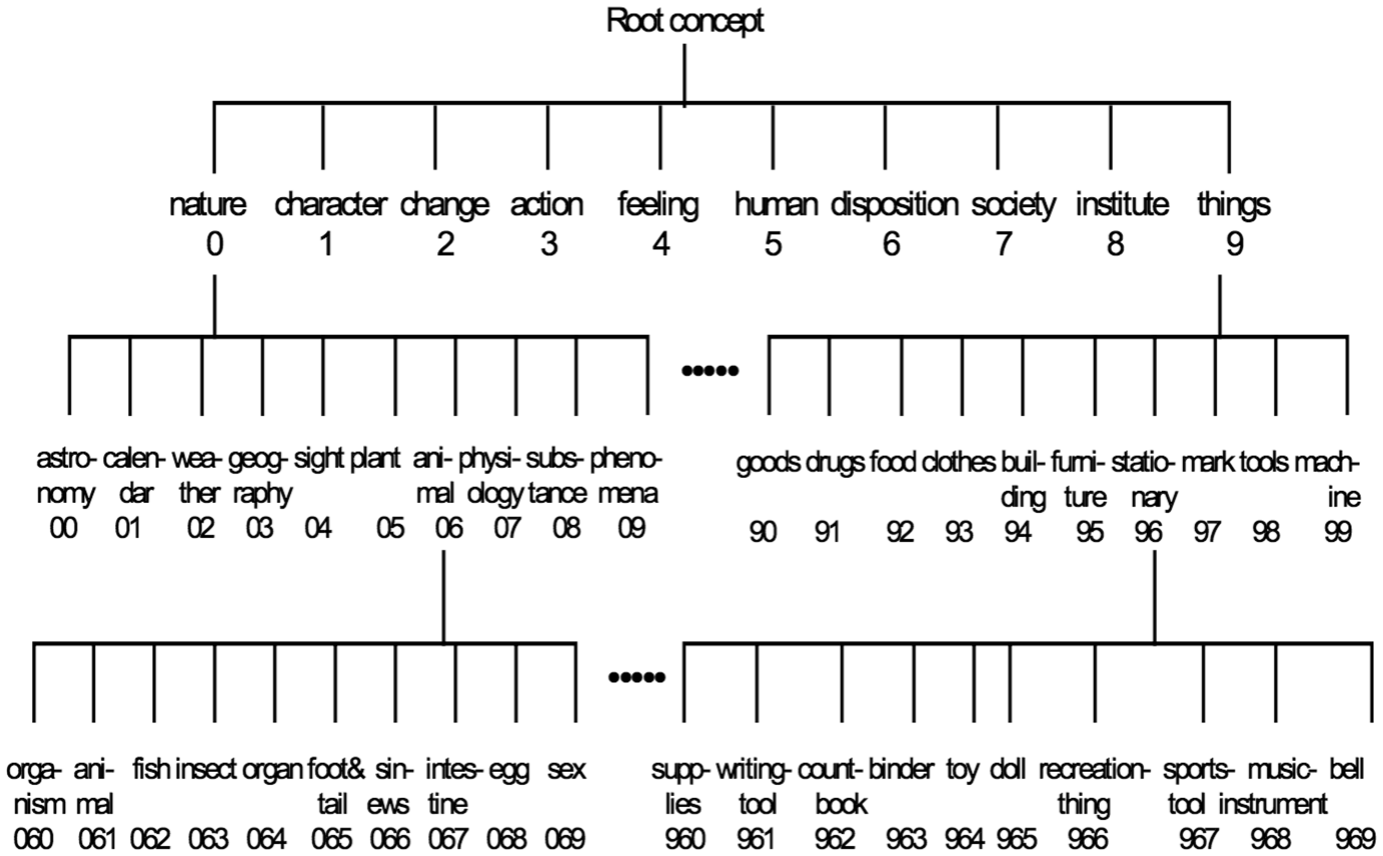
Kadokawa Thesaurus Hierarchy [39]

### Answering Yes/No Questions—Tasks 4 and 5

#### Tasks Definition

Task 4 is a task to determine textual entailment relationships between a given problem sentence and relevant article sentences. Competitor systems should answer “yes” or “no” regarding the given problem sentences and given article sentences. Task 5 requires a system to answer “yes” or “no” given a problem sentence(s) only. Participants can use any external data; however, this assumes that they do not use the test dataset.

#### Approach

The problem of answering a legal yes/no question can be viewed as a binary classification problem. Assume a set of questions *Q*, where each question $q_i \in Q$ is associated with a list of corresponding article sentences ${a_{i1}, a_{i2},\ldots , a_{im}}$, where $y_i = 1$ if the answer is ‘yes’ and $y_i = 0$ otherwise. We choose the most relevant sentence $a_{ij}$ using the algorithm of Kim et al. [2], and we simply treat each data point as a triple $(q_i, a_{ij}, y_i)$. Therefore, our task is to learn a classifier over these triples so that it can predict the answers of any additional question–article pairs. BERT [9] has shown good performance on the general natural language inference task. However, Jiang and Marnaffe [26] insisted that despite high F1 scores, BERT models have systematic error patterns, suggesting that they do not capture the full complexity of human pragmatic reasoning. To aid the pragmatic reasoning, our system incorporates the semantic information into the BERT language model for natural language inference.

The entailment result based on the syntactic parser, article segmentation and negation detection showed the best performance in COLIEE 2019. We will call this approach *SYN*. However, in COLIEE 2020, BERT showed better performance than *SYN*. In COLIEE 2021, we combine these two approaches to achieve synergy. We see some cases where the prediction output of BERT is different from the output of *SYN*. To resolve the different prediction issue between the two systems, we use additional information, which is semantic closeness. We add this semantic analysis component using the syntactic parser of Kim et al. [40].

To measure semantic closeness, we use semantic category codes of the Kadokawa thesaurus corresponding to the content words in the input, as shown in Fig. 2. In COLIEE 2021, we compare the entailment outputs between BERT and *SYN*. If they are the same, the entailment output is adopted with consensus. Otherwise, we check the semantic codes of the Kadokawa thesaurus of the content words in the query and the relevant article. Then we just apply the following simple heuristic rule:

- If there are shared semantic codes in both of the following cases: (1) between the conditions of the query and the article, and (2) between the conclusions of the query and the article, then we choose the answer “yes.” Otherwise, the answer is “no.”

**Fig. 3:**
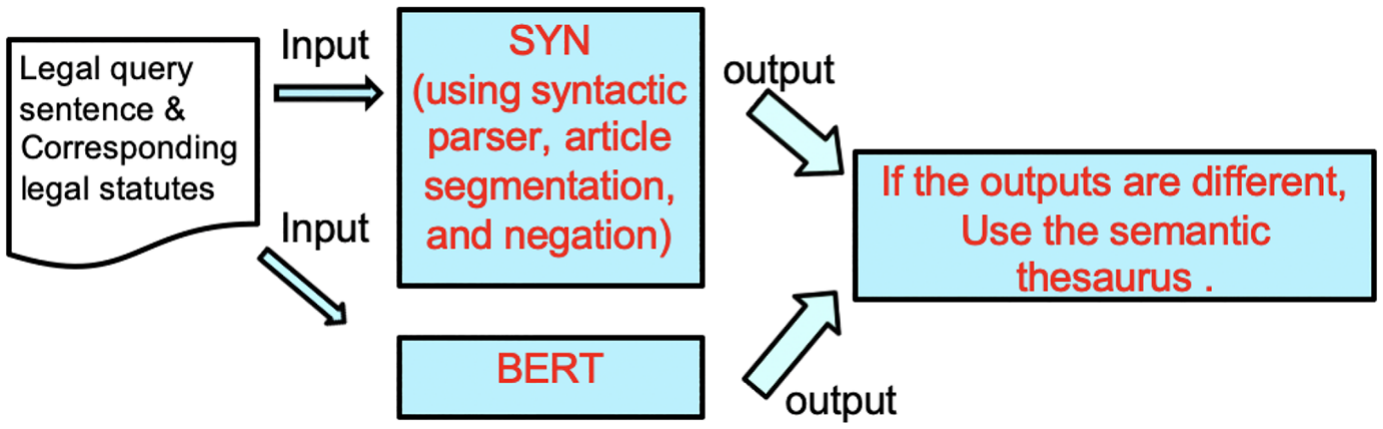
Architecture of Task 4

Figure 3 shows the architecture of the Task 4 model.

Following is one example:

*Query: A, who acts(code:754,822) as the agent(code:552) of B, concluded(code:448) a contract(code:448) with C for sale(code:742) of land(code:042) owned(code:379) by B. However, A did not possess the authority(code:449) to conclude the contract(code:448). If B ratifies(code:444) the contract(code:448) of sales, A is not liable(code:449) to C as an unauthorized agency(code:552).*

*Relevant Article: A person(code:507) who concludes a contract(code:448) as an agent(code:552) of another person is liable(code:449) to the counterparty(code:505) for the performance of the contract or compensation(code:375) for loss(code:744) or damage(code:744), as chosen by the counterparty, unless the person proves(code:418,817) the authority(code:449) to represent(code:552) or the principal(code:505) ratifies(code:444) the contract.*

For the above example, the output of BERT was “yes,” but the output of *SYN* was “no.” So we then check if there are any content words that share the same semantic code in the conditions of the query and the article. We do the same check for the conclusions. In this example, there are content words that share the same semantic codes. Therefore, the output of our system will be “yes.”

Table 3 shows the Task 4 results on test data in COLIEE 2021. In the table, UA_parser is the system combining pre-trained BERT fine-tuned on the SNLI^4^ dataset and semantic code with the syntactic parser. We also used ELMo-based decomposable attention trained on the SNLI dataset [41] (name: UA_Elmo), and RoBERTa [42] fine-tuned on the SNLI dataset (name: UA_dl). We did not achieve better performance when we fine-tuned models on COLIEE training data, so we fine-tuned our models on the SNLI dataset.

UA_parser is the only system that incorporates the semantic information (Kadokawa thesaurus concept code), and it was ranked no. 4 in Task 4 of COLIEE 2021.

**Table 3:** NLI (Task 4) results on test data

Team	Sid	Correct	Accuracy
	BaseLine	43/All 81	0.5309
HUKB	HUKB-2	57	0.7037
HUKB	HUKB-1	55	0.6790
HUKB	HUKB-3	55	0.6790
UA	UA_parser	54	0.6667
JNLP	JNLP.EC	51	0.6296
JNLP	JNLP.ECS	51	0.6296
JNLP	JNLP.EB	51	0.6296
OVGU	OVGU_run3	48	0.5926
TR	TR-Ensemble	48	0.5926
TR	TR-MTE	48	0.5926
OVGU	OVGU_run2	45	0.5556
KIS	KIS1	44	0.5432
KIS	KIS3	44	0.5432
UA	UA_elmo	44	0.5432
KIS	KIS2	43	0.5309
UA	UA_dl	43	0.5309
TR	TR_Electra	41	0.5062
OVGU	OVGU_run1	36	0.4444

The difference between Task 4 and Task 5 is whether or not the gold standard answer for the relevant statutes is used. In Task 4, participants use the gold standard for relevant statutes provided by the organizers, while in Task 5, participants use the retrieved statutes using their own results of Task 3. Table 4 shows the results of the submitted systems in COLIEE 2021 for Task 5. When we submitted the Task 5 results, we did not know that our BM25 technique showed the best performance amongst our three submitted systems in Task 3. So, we chose the output of a traditional TF-IDF technique for IR, and combined the IR output with our NLI systems for Task 5 submission. Our system combining TF-IDF in IR (Task 3) + UA_parser (Task 4) was ranked no. 2. As future work, we will combine our NLI approach with our BM25 technique and see if it can improve our current Task 5 performance.

**Table 4:** Task 5 (IR+NLI) results on test data in COLIEE 2021

Team	Sid	Correct	Accuracy
	BaseLine	43/All 81	0.5309
JNLP	JNLP.NFSP	49	0.6049
UA	UA_parser	46	0.5679
JNLP	JNLP.NMSP	45	0.5556
UA	UA_dl	45	0.5556
TR	TRDistillRoberta	44	0.5432
KIS	KIS_2	41	0.5062
KIS	KIS_3	41	0.5062
UA	UA_elmo	40	0.4938
JNLP	JNLP.task5.B_M	38	0.4691
KIS	KIS_1	35	0.4321
TR	TRGPT3Ada	35	0.4321
TR	TRGPT3Davinci	35	0.4321

### Error analysis in Statute Law Entailment

From unsuccessful instances in Task 4, we classified the error types as shown in Table 5. The biggest error arises, of course, from the paraphrasing problem. For example, machines were not able to identify that “obligee” = “beneficiary.” One interesting thing is that UA_elmo and UA_dl have many cases of negation-related errors while UA_parser has only one case. We believe this is because the syntactic analyzer can correctly identify the boundary of the negation through syntactic dependency analysis. We can also see that many errors arise from the complex constraints in condition and conclusion. In addition, there are many cases belonging to the reference resolution error. For example, there is a query *“A, who acts as the agent of B, concluded a contract with C for sale of land owned by B.”*. In Task 4, machines should be able to identify what A, B, and C are referring to in the relevant article. Currently, in analyzing this kind of input including the reference terms, some errors occurred. At the current stage, we do not employ any specific reference resolution process that can deal with this kind of complicated input, but just rely on BERT and *SYN* and get the final prediction outcome. If we do not have an appropriate reference resolution process, we will not be able to consider that the system semantically understands the input sentences. As future work, we need to figure out how these reference terms can be correctly resolved, in order to get the correct prediction outcome based on *real* understanding of the input sentences. The current data samples that have the referring terms such as *A, B,* and *C* are hard to be understood by machines because these terms can be *examples* of the relevant article case. This is an open challenge that we need to consider in future work.

**Table 5:** Task 4 Error types

Error type	UA_parser	UA_dl	UA_elmo
Wrong analysis of condition	7	9	6
Wrong analysis of conclusion	1	1	1
Negation detection error	1	5	9
Paraphrase detection error	15	15	16
Reference resolution error	1	3	3
Wrong analysis of conjunction	1	2	1
etc.	1	3	1

## Conclusion

We explained our models for legal entailment and question answering in COLIEE 2021. For the case law entailment task, our transformers-based system ranked 4th place among all submissions (2nd among all teams). Our future work will include exploring combinations of complementary techniques as well as alternatives for appropriate data augmentation for Task 2. We have experimented with data augmentation in the past but without much success (please see [2] for more details). However, we believe we can produce better results if we can find alternative data sources. For the statute law tasks, our BM25 system was ranked 3rd in Task 3, and our NLI system combining BERT and semantic information was ranked 4th in Task 4 (we were the 2nd best team in that task) and 2nd in Task 5. As future research, we will investigate methods to obtain semantic representation for paragraphs and perform natural language inference between paragraphs.

## Footnotes

1 Calling the kind of representations learned by BERT (or any other transformer-based model) “knowledge” is a stretch and even some sort of anthropomorphization but seems to be appropriate in the context of machine “learning.”

2 https://pypi.org/project/langdetect/.

3 https://en.wikipedia.org/wiki/Okapi_BM25.

4 https://nlp.stanford.edu/projects/snli/
